# Using ecological observations to improve malaria control in areas where *Anopheles funestus* is the dominant vector

**DOI:** 10.1186/s12936-022-04198-3

**Published:** 2022-06-02

**Authors:** Najat F. Kahamba, Marceline Finda, Halfan S. Ngowo, Betwel J. Msugupakulya, Francesco Baldini, Lizette L. Koekemoer, Heather M. Ferguson, Fredros O. Okumu

**Affiliations:** 1 Environmental Health and Ecological Sciences Department, Ifakara Health Institute, P. O. Box 53, Ifakara, Tanzania; 2 Institute of Biodiversity, Animal Health and Comparative Medicine, University of Glasgow, G128QQ Glasgow, UK; 3 School of Public Health, Faculty of Health Science, University of the Witwatersrand, Johannesburg, South Africa; 4 Department of Vector Biology, Liverpool School of Tropical Medicine, Liverpool, UK; 5 Wits Research Institute for Malaria, School of Pathology, Faculty of Health Sciences, University of the Witwatersrand, Johannesburg, South Africa; 6 School of Life Science and Biotechnology, Nelson Mandela African Institution of Science and Technology, P. O. Box 447, Arusha, Tanzania

**Keywords:** Malaria transmission, Vector ecology, Larval source management, ITNs, IRS, Ifakara

## Abstract

The most important malaria vectors in sub-Saharan Africa are *Anopheles gambiae, Anopheles arabiensis, Anopheles funestus*, and *Anopheles coluzzii*. Of these, *An. funestus* presently dominates in many settings in east and southern Africa. While research on this vector species has been impeded by difficulties in creating laboratory colonies, available evidence suggests it has certain ecological vulnerabilities that could be strategically exploited to greatly reduce malaria transmission in areas where it dominates. This paper examines the major life-history traits of *An. funestus*, its aquatic and adult ecologies, and its responsiveness to key interventions. It then outlines a plausible strategy for reducing malaria transmission by the vector and sustaining the gains over the medium to long term. To illustrate the propositions, the article uses data from south-eastern Tanzania where *An. funestus* mediates over 85% of malaria transmission events and is highly resistant to key public health insecticides, notably pyrethroids. Both male and female *An. funestus* rest indoors and the females frequently feed on humans indoors, although moderate to high degrees of zoophagy can occur in areas with large livestock populations. There are also a few reports of outdoor-biting by the species, highlighting a broader range of behavioural phenotypes that can be considered when designing new interventions to improve vector control. In comparison to other African malaria vectors, *An. funestus* distinctively prefers permanent and semi-permanent aquatic habitats, including river streams, ponds, swamps, and spring-fed pools. The species is therefore well-adapted to sustain its populations even during dry months and can support year-round malaria transmission. These ecological features suggest that highly effective control of *An. funestus* could be achieved primarily through strategic combinations of species-targeted larval source management and high quality insecticide-based methods targeting adult mosquitoes in shelters. If done consistently, such an integrated strategy has the potential to drastically reduce local populations of *An. funestus* and significantly reduce malaria transmission in areas where this vector species dominates. To sustain the gains, the programmes should be complemented with gradual environmental improvements such as house modification to maintain biting exposure at a bare minimum, as well as continuous engagements of the resident communities and other stakeholders.

## Background

For the past twenty years, there has been increased international focus on improving malaria control and accelerating efforts towards elimination [1]. Significant progress was made until 2015, mainly due to the scale-up of effective vector control interventions including insecticide-treated nets (ITNs) and indoor residual spraying (IRS). Universal coverage of these interventions coupled with effective case management contributed most of the gains [2]. Yet the impact of these interventions appears to be flattening in sub-Saharan Africa, where malaria accounts for 95% of cases and 96% of deaths [1]. Further progress with these existing core vector control interventions (ITNs and IRS) is now limited by various mosquito adaptations notably resistance to public health insecticides, behavioural adaptations [3, 4]. Other challenges include low-level funding for malaria and general weaknesses in the health systems.

In addition to the constraints generated by evolutionary adaptations and socio-economic factors, the impact of vector control is hindered by ecological heterogeneity in how vectors, parasites, and human hosts interact with one another and the environment [5]. For instance, different vector species require different ecological conditions to complete vital life cycle processes such as oviposition, larval development, mating, and blood-feeding. Specifically, vector species may vary in their use and preference of sugar sources, hosts, larval habitats, or resting sites [6].

Unfortunately, such species-specific differences are rarely considered when implementing vector control, with the two core interventions of IRS and ITNs being similarly recommended for all the major African vector species and across most settings [1]. This “one size fits all” approach may simplify the deployment and scale-up vector control programmes, but it is erroneous to assume that all vector species are vulnerable and respond similarly to these and other interventions [7]. For example, indoor interventions such as ITNs and IRS are very effective against mosquitoes that mostly bite humans indoors and also rest indoors, but are less effective against exophilic and zoophagic populations [8, 9]. Given the increasing recognition of the role of outdoor-biting, outdoor-resting and zoophagic species in maintaining residual transmission [8], it is important that interventions target all relevant ecological and behavioural adaptations of key vector species [7].

The major malaria vectors in sub-Saharan Africa (SSA) include *Anopheles coluzzii, Anopheles gambiae sensu stricto (s.s.), Anopheles funestus s.s.*, and *Anopheles arabiensis*, but several others also play secondary role in specific localities [10]. These vector species differ in bionomics, vectorial capacities, and contribution to overall transmission, resulting in varying stability of malaria transmission across geographies [11]. The importance of *An. funestus s.s.* (hereafter is referred to simply as *An. funestus)* as a dominant malaria vector has been documented in many east and southern African countries [12–17]. In locations such as south-eastern Tanzania [12, 18], and in some districts in northern Tanzania around Lake Victoria [19], this species is responsible for 85–97% of all malaria transmission events. In addition to having relatively high sporozoite prevalence and high vectorial capacity, *An. funestus* has also been shown to be highly resistant to insecticide [19], long survival [20], and more anthropophilic [21] than co-existing vector species in several settings. Consequently, *An. funestus* may have among highest vectorial capacity of all African vector species.

The disproportionate role of *An. funestus* reflects the basic Pareto distribution, with most of the transmission coming from this species even in areas where it has relatively lower abundance in the overall vector community [22]. The dominance of *An. funestus* as a vector suggests that prioritizing the species for control may yield significant suppression or even local elimination of transmission in the respective settings [12]. More targeted strategies against *An. funestus* would require an improved understanding of the biology and ecology of the species, which remains challenging and relatively neglected due to the complexities of studying this species in the laboratory and in the wild [23, 24]. Together with the difficulties in creating laboratory colonies of the species, the above constraints have led to major knowledge gaps. These gaps are often bridged in intervention or modeling studies by assuming that information from other African vectors, for example *An. gambiae sensu lato* (*s.l.*), are broadly transferrable to *An. funestus*.

This article challenges this assumption of generalizability with other African vector species by synthesizing the existing knowledge on the life history, behaviour, and ecology (larval and adult) of *An. funestus*. The article highlights key knowledge gaps in the current understanding of this species and highlight areas of its ecology that may generate differential responsiveness to key interventions. Based on these insights, plausible strategies are presented for significantly disrupting malaria transmission in areas where *An. funestus* dominates through the implementation of combined interventions tailored to its ecology.

### **Distribution and importance of*Anopheles funestus*in the east and southern Africa**

The *An. funestus* group consists of at least 11 known African species whose distribution extends across sub-Saharan Africa [10]. The members of this group include *Anopheles funestus* (*s.s*.), *Anopheles vaneedeni*, *Anopheles parensis*, *Anopheles aruni*, *Anopheles confusus*, *Anopheles rivulorum*, *Anopheles fuscivenosus*, *Anopheles leesoni*, and *Anopheles brucei* [25, 26]. Additional species recently included are *An. funestus*-like, which were identified in Malawi [27] and *An. rivulorum*-like, identified in Cameroon [26, 28]. Other studies from different locations suggest a further subdivision of *An. funestus* into three geographically distinct molecular types (M, W, MW), with the M- type found in eastern Africa, W in western and central Africa and MW present in southern Africa [29]. However, more than one molecular form has been reported in some locations [29]. For example, all three types have been found in Malawi, both M and MW-types in Tanzania, and the M and W-type in Kenya [29]. Furthermore, recently two more types have been described: Y from Malawi and type Z from four locations of Angola, Malawi, Ghana, and Zambia [30].

The sibling species in the *An. funestus* group appear to have different biology and a role in malaria transmission. They are also morphologically similar at the adult stage, making differentiation difficult thus requiring molecular identification [31]. Although highly-skilled taxonomists can separate species based on immature aquatic stage morphology [32, 33]. Given the limited capacity for molecular identification in many settings, many members in the group can be easily be misidentified [25], potentially leading to the potential role of other species within the funestus group being misunderstood.

However, to date *An. funestus* remains the most significant vector in this group. Data from east Africa, where *An. funestus* is now highly resistant to common public health insecticides [34], indicates very high sporozoite infection rates compared to other *Anopheles* vector species [12, 19]. In these locations, it is evidently responsible for most of the transmission as measured by entomological inoculation rates (EIR). Higher infection prevalence has also been reported in Zambia [35], Malawi [13], and the Islands of Madagascar [36]. Beyond East and southern Africa, *An. funestus* is also an important vector in Central and West Africa. In west African countries such as Ghana [37], Côte d’Ivoire [38], and Benin [39], *An. funestus* has been reported alongside other species such as *An. gambiae* and *An. coluzzii*. Table 1 provides examples of selected studies from different African countries, where the species has been investigated, and its importance in malaria transmission described. These studies broadly show that *An. funestus* typically has among the highest infections rates (Table 1).

**Table 1:** Examples of some studies in Africa showing the role of different *Anopheles* species in malaria transmission

SN	Country	Year	Dominant vector[s]	Other *Anopheles*	Sporozoite prevalence	EIR contribution	Feeding habits	Human blood index	Resistance status and mechanism of resistance detected	Refs.
1	Tanzania	2021	*An. funestus*	*An. arabiensis* *An. parensis*, *An. rivulorum*, *An. gambiae s.s*	Not reported	*An. funestus s.l* (96.47%) *An. gambiae s.l* (3.53%)	*An. funestus*: endophilic *An. arabiensis* exophilic	Not reported	*An. funestus*: resistant to pyrethroids L1014S-*Kdr mutation detected in An. gambiae s.s.*	[1]
2.	Tanzania	2018	*An. funestus*	*An. arabiensis* *An. coustani*	*An. funestus* (0.205%) *An. arabiensis* (0%)	*An. funestus* (100%) *An. arabiensis* (0%)	Not reported	Not reported	*An. arabiensis* confirmed resistance toward pyrethroid	[2]
3.	Zambia	2017	*An. funestus*	*An. leesoni* *An. gambiae s.s*	*An. funestus* (2.7%) *An. gambiae s.s* (3.1%)	*An. funestus* (87.03%) *An. gambiae s.s* (19.97%)	Not reported	*An. funestus* (3.2%) *An. gambiae s.s*, (25.7%)	Not reported	[3]
4.	Tanzania	2017	*An. funestus*	*An. arabiensis* *An. leesoni* *An. rivulorum* *An. pharoensis* *An. squamosus* *An. ziemanni* *An. wellcomei*	*An. arabiensis*, (0.0002%) *An funestus*, (0.0053%)	*An. funestus* (86.21%) *An. arabiensis* (13.79%)	*An. funestus* anthropophagic	*An. funestus (*100%) *An. leesoni* (100%) *An. arabiensis* (73.4%)	*An. funestus* resistance to deltamethrin, permethrin, lambda cyhalothrin and DDT confirmed Susceptible to Pirimiphos-methyl, malathion and dieldrin	[4]
4.	Kenya	2011	*An. funestus s.l.*	*An. gambiae s.s* *An. arabiensis*	*An. funestus* (0.0057%) *An. gambiae* (0.0043%)	*An funestus s.l* 63.6% *An. gambiae s.l* 36.3%	Not reported	*An funestus s.l* (94.1%) *An. gambiae* (83.9%)	Not reported	[5]
5.	Madagascar	2010	*An. funestus*	*An. gambiae* *An. mascarensis*	*An. funestus* (1.58%) *An. gambiae* s.l. (0.48%) An. mascarensis (0.75%)	*An. funestus* (77.3%) *An. gambiae* (6.47%) *An. mascarensis* (16.19%)	*An. funestus* Anthropophagic *An. gambiae* Anthropophagic	Not reported	Not reported	[6]
6.	Kenya	2017	*An. funestus*	*An. arabiensis* *An. gambiae s.s* *An. coustani* *An. pharoensis*	*An. funestus* (1.8%) *An. arabiensis* (0.16%)	*An. funestus* (63.6%) *An. arabiensis* (36.3%)	*An. funestus* anthropophagic *An. arabiensis* exophilic and zoophagic	*An. funestus* (60%) *An. arabiensis* (2.5%) *An. gambiae* (50%)	Not reported	[7]
7	Benin	2019	*An. arabiensis*	*An. funestus s.s* *An. coluzzii* *An. gambiae s.s* *An. ziemani* *An. pharaonis*	*An. funestus* (0.048%) *An. gambiae* s.l (0.017%) *An. nilli* (0.0125%)	*An. funestus* (5.86%) *An. gambiae* s.l (82.2%) *An. nilli* (11.9%)	Not reported	*An. gambiae* s.l (91.3%)	Not reported	[8]
8.	Rwanda	2018	*An. gambiae*	*An. funestus* *An. ziemanni* *An. coustani*	*An. gambiae* s.l (2.79%)	*An. gambiae* s.l (100%)	*An. gambiae* s.l endophily	Not reported	Not reported	[9]
9.	Ethiopia	2017	*An. arabiensis*	*An.funestus* s.l, *An. demeilloni* *An. cinereus*, *An. pharoensis*,	*An. arabiensis* (3%) *An. demeilloni*, (0%)	*An. arabiensis* (100%) *An. demeilloni* (0%)	Not reported	Not reported	Not reported	[10]
10.	Ethiopia	2017	*An. arabiensis*	*An. funestus s.l.* *An. coluzi* *An. pharoensis*	*An. funestus* (2.3%) *An. arabiensis* (4.1%) *An. pharoensis* (4.5%)	*An. funestus* (22.6%) *An. arabiensis* (61.5%) *An. pharoensis* (15.7%)	*An. funestus* Anthropophagic *An. arabiensis* Anthropophagic	*An. funestus* s.l. (87.2%) *An. arabiensis* (82.4%)	Not reported	[11]
11.	Côte d’Ivoire	2015	*An. gambiae*	*An. funestus* *An. nilli* *An. pharoensis* *An. coustani* *An. ziemanni* *An. wellcomei* *An. brohieri*	*An. funestus* (1.3%) *An. gambiae* (2.5%)	*An. funestus* (7.85%) *An. gambiae* (92.15%)	Not reported	Not reported	Not reported	[12]
13.	Ghana	2012	*An. gambiae s.s*	*An. arabiensis*, *An. funestus* *An. pharoensis*	*An. gambiae s.s.* (1.52%) *An. funestus* (0%)	*An. gambiae* s.s. (100%) *An. funestus* (0%)	Not reported	*An. gambiae* s.s. (66.67%)	Not reported	[13]
14.	Chad	2009	*An. arabiensis*	*An. pharoensi* *An. funestus* *An. ziemann*	*An. arabiensis* (1.4%) *An. funestus* (1.4%) *An. pharoensi* (0.8%) *An. ziemann* (0.5%)	*An. arabiensis* (84.5%) *An. pharoensis* (12.2%) *An. funestus* (2.5%) An. ziemanni (0.8%)	*An. arabiensis*, endophagic *An. funestus* endophagic	*An. funestus* (90.6%) *An*. *pharoensis* (71.4%) *An. arabiensis* (63.9%)	Not reported	[14]
15.	Cameroon	2005	*An. gambiae*	*An. moucheti* *An. funestus*	*An. gambiae* (15.3%) *An. moucheti* (3.4%) *An. funestus* (17.0%)	*An. gambiae* (84%) *An. moucheti* (11%) *An. funestus* (5%)	Not reported	Not reported	Not reported	[15]
16.	Nigeria	2010	*An. gambiae s.s*	*An. melas* *An. nilli*	*An. gambiae s.s (42.5%)* *An. melas (57.5%)* *An. nili (0%)*	*An. gambiae s.s (83%)* *An. melas (17%)* *An. nili (0%)*	Not reported	*An. gambiae s.s (63.3%)* *An. melas (73.8%)* *An. nili (0%)*	Not reported	[16]

*N.B These papers were randomly selected as examples to show the reported importance of An. funestus in malaria transmission in different settings in Africa. The search was done intentionally to provide examples of reported importance in different setting*

Many other species in the *An. funestus* group are not known to be malaria vectors. However, *An. rivulorum* has been incriminated in some locations in Tanzania and Kenya [12, 40, 41]. In South Africa, both *An. vaneedeni* and *An. parensis* have been shown to contribute to residual malaria transmission [42]. Another study in Kenya did not provide evidence of *An. parensis* supporting transmission, although this species was commonly found resting indoors, it was mainly feed on cows and uninfected with malaria parasites [43]. In South Africa, indoor densities of *An. parensis* outnumbered *An. funestus* following extended IRS campaigns [42] and thus, their role in sustaining residual malaria transmission needs to be determined. Another member of *An. funestus* group previously incriminated in transmission was *An. leesoni* in eastern Tanzania [44]. Overall, there are very limited investigations of these other sibling species or their involvement in malaria transmission, and rarely they are identified or screened during routine entomological surveillance.

### **Larval ecology of*Anopheles funestus***

Even though there has only been a small number of studies that specifically focused on the larval habitats of *An. funestus*, there are several field investigations that have revealed that *An. funestus* larvae can co-exist with other malaria vectors [45]. In the early work done in the 1930s, *An. funestus* was observed to breed in clear permanent water bodies including swamps, streams, ditches and ponds [46]. Aquatic habitats containing the larvae were characterized as being shaded by hanging trees, bushes, or emergent vegetation [46]. Another early study from Malindi in the east coast of Kenya reported the rare occurrence of *An. funestus* as a domestic mosquito breeding in wells and domestic water containers [47].

A distinct feature of *An. funestus* larval ecology is that this species typically occupies larger and more permanent or semi-permanent water bodies than other malaria vectors; often characterized with emergent or floating vegetation [46]. These habitats generally do not have direct sunlight exposure [46]. *Anopheles funestus* is indeed rarely found in completely open waters or in small sunlit puddles [61], contrary to other African vector species, such as *Anopheles arabiensis* and *An. gambiae*, which frequently use small or temporary habitats such as footprints [49, 50]. The differential use of larval habitats has been associated with seasonality in malaria transmission patterns, with *An. gambiae s.l.* driving the large transmission peaks in the rainy season, while *An. funestus* being more able to sustain high levels of malaria transmission throughout the year [12]. Indeed, field observations in eastern Africa have shown that the adult population of this species often peak shortly after the rains [12, 51].

**Fig. 1:**
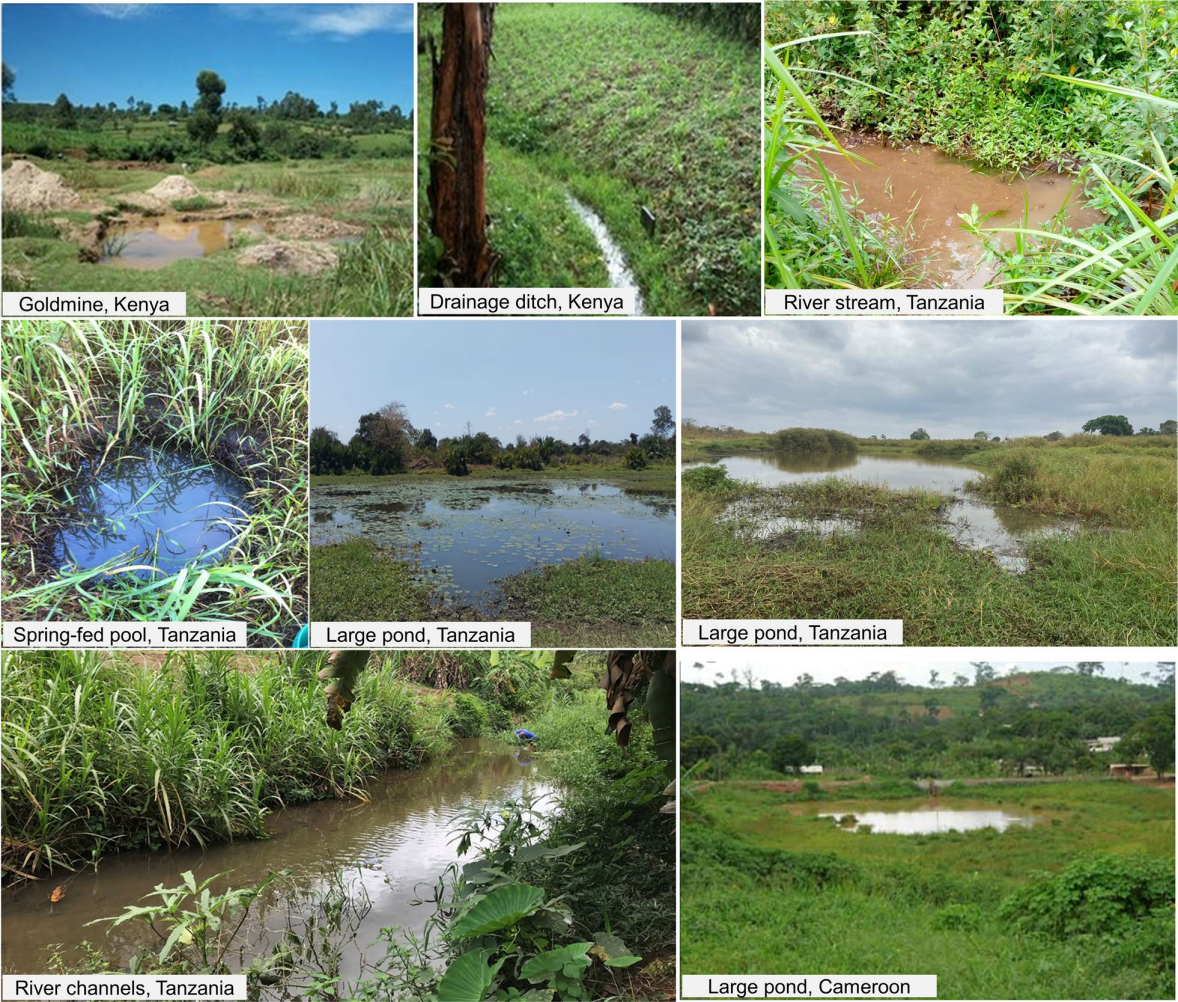
Examples of common aquatic habitat types for *Anopheles funestus* in Kenya, Cameroon, and southern Tanzania. Pictures were adapted from published articles by Kweka et al. [49] and Nambunga et al. [46]

The permanent habitats of *An. funestus* include slow-moving waters along the edges of rivers, especially on tributaries found on rising altitudes [46, 52]. In Tanzania, Nambunga et al. [46] categorized larval habitats used by *An. funestus* into 3 types: (i) small ponds and spring-fed wells found at low altitudes (150–200 m), (ii) slow-moving waters along rivers and streams at higher altitudes above 300 m, and (iii) large open ponds that maintain water for most of the year in both low and high altitude areas. The most prolific of these habitats were the rivers and streams [46]. Elsewhere in east Africa, *An. funestus* has also been observed breeding in lakeshore pools during periods of low water [53], while in west Africa this species has mostly been described as breeding in river tributaries [54] (Fig. 1). These larval habitat descriptions are mostly specific to *An. funestus.* However, other sibling species such as *An. rivulorum, An. leesoni*, and *An. parensis* have been observed to share aquatic habitats with *An. funestus* [31], though there can be differences in their level of tolerance to salinity [55]. Consequently, larval source management (LSM) targeted *An. funestus* could potentially also impact other secondary vector species in this group.

The overall survival and development of *Anopheles* larvae are influenced by several biotic and abiotic factors including the availability of nutrients, larval densities, and predation [56]. For instance, mosquito larvae developing in crowded habitats often have reduced body size, as well as reduced lipid, glycogen, and protein contents due to increased intra-specific competition for resources [57]. Larval development is also very sensitive to climatic conditions; with varying sensitivity to temperatures and rainfall [58] as well as salinity [55]. In particular, *An. funestus* larvae tend to be more sensitive to fluctuations in water temperatures than other vector species [59], which partly explains why the species often occupies larger perennial habitats with minimal microclimate fluctuations [58, 59]. The optimum temperature for *An. funestus* is 27 °C, however survival declines when temperature approach 32 °C and lower to 18 °C. Rainfall tend to refill habitats and perpetuates vector populations whereas the cumulative lag (two weeks) rainfall increases survival. However, excessive downpours and flooding can destroy habitats and flush out the larvae, eggs, and pupae [24].

### **Adult ecology of*Anopheles funestus***: **behaviour, important life-history traits, and survival strategies**

The behaviours of adult *Anopheles* have a direct impact on their vectorial capacity, a measure that describes the transmission potential of a vector in terms of its abundance, survival, ability to transmit pathogens and rate of feeding on humans [60]. Vector species that adapted to specialize on humans are more efficient transmitters of human malaria than those with opportunistic or generalist feeding behaviours [61]. *Anopheles funestus* is usually highly endophilic (refers to a tendency of indoor resting) and anthropophilic (refers to a tendency of feeding on humans), giving rise to its high vectorial capacity amongst African vectors [21]. Field records of the proportion of blood meals that mosquitoes obtain from humans as opposed to other vertebrates, i.e., the human blood index (HBI) suggest that *An. funestus* and *An. gambiae s.s.* have the highest HBI values among African malaria vectors. This explains their competency as vectors of malaria, and the stability of malaria in tropical Africa where these species are present [11, 62].

With regard to their blood-feeding and resting habits, *An. funestus* is often assumed to be most similar *An. gambiae s.s.* [63], but there are specific instances where this species has been reported biting outdoors [51], resting outdoors [68], and being attracted to cattle [64, 65]. Modest levels of zoophagy have been documented in some cattle-keeping communities [61]. As molecular identification was not performed to confirm species identity in past literature, other morphologically cryptic species within the *An. funestus s.l.* might be responsible for these reports of exophily and zoophily. Consequently, the existence and potential importance of outdoor biting in this species may have been underexplored and may need to be updated. For example, *An. rivulorum* is a species that is morphologically similar to *An. funestus*, but more associated with exophilic and endophilic behaviours [40]. However, in the most recent study, after molecular characterization, it was confirmed that *An. funestus* were attracted to both humans and cattle [65], suggesting that some degree of zoophagy may occur in this species [64].

*Anopheles funestus*, like other *Anopheles* species, mates in aerial swarms. In comparison to *An. gambiae s.l.* the swarms of *An. funestus* tend to be smaller and more difficult to locate [66, 67]. *Anopheles funestus* is refractory to mating in confined spaces, and instead appear to require large open spaces to mate [6, 68]. In Tanzania [66] and Mozambique [69], where *An. funestus* swarms have been characterized, males were observed to congregate close to human dwellings inside villages, unlike swarms of *An. arabiensis* that are generally found at the edges of the village. While *An. funestus* is thought to primarily mate outdoors, new evidence indicates that significant proportions of mating in both *An. funestus* and *An. arabiensis* can occur inside homes [70], corroborating previous observations of *An. gambiae s.l.* mating inside experimental huts in west Africa [71]. While the ecological significance of such indoor mating remains to be elucidated, the observation of large densities *An. funestus* males resting inside houses suggests it might be a common occurrence [70]. Furthermore, because of the apparent high degree of eurigamy, inducing mating in the laboratory is very difficult. As a result, there have been relatively few successful efforts to colonize *An. funestus*, with just two well-established colonies in existence from Angola (FANG) [72] and Mozambique (FUMOZ) [73]. Given the complexity associated with mating behaviours, further research should be conducted to address this challenge [23]. There are currently ongoing attempts in Tanzania towards these objectives, though this has initially focused on assessing key fitness and survival parameters of *An. funestus* [23, 24].

The survival of adult female mosquitoes is a crucial determinant for their vector capacity since the mosquito must survive for at least 10–12 days to be able to transmit malaria parasites [6]. Unfortunately, direct measurement of adult mosquito survival in the field are difficult, and only a small number of methods are available to estimate through indirect measures such as mark-recapture or ovarian dissection [6]. Such estimates can vary depending on factors such as variations in the technical skill of the personnel and the widespread use of insecticidal interventions such as ITNs in the field. Nonetheless, the limited amount of available evidence suggests that *An. funestus* has greater adult survival than other malaria vectors such as *An. arabiensis* [69, 74]. In Tanzania, the daily survival probabilities estimated before wide-scale ITNs use were consistently greater than 80% [75]. More recent estimates of age structure based on parity dissections suggest *An. funestus* survival is greater than *An. arabiensis* in some settings [76]. This greater longevity of *An. funestus* and combination with anthropophilic behaviours provide multiple opportunities for this vector to become infected and transmit malaria.

Lastly, changes in climatic conditions may also have a substantial influence on the survival and longevity of *An. funestus*. For instance, very low and high temperatures influence their development and survival [77]. Unfortunately, there has been little research examining the direct effect of temperature on *An. funestus* life-history characteristics.

### **Exploiting the ecology of*Anopheles funestus*to improve malaria control in areas where the species dominates**

#### Larval source management (LSM)

There are four main strategies for LSM; (1) habitat modification; refers to alterations made to the environments to limit vector breeding, (2) habitat manipulation; refers to repeated activities that remove the larvae, such as flushing streams, (3) larviciding; refers to regular application of insecticides to water bodies where mosquitoes breed, and (4) biological control; refers to the introduction of natural predators such as larvivorous fish into aquatic habitats. The suitability of each approach depends on the local ecology of the main malaria vector, as well as the environmental conditions. For example, the temporary, small, and scattered larval habitats of *An. gambiae s.s.* could perhaps be simply dried up, covered, or removed (i.e., habitats modification). On the other hand, the larger, more permanent habitats used by *An. funestus* (e.g., large ponds and streams) may be suitable for direct environmental modification and manipulation.

There may however be some notable challenges for the control of *An. funestus* in aquatic habitats. For example, the spring-fed pools used by the species may also be a source of clean water for local communities. Thus, removal of these habitats would not be appropriate. Instead, specific larvicides that pose no safety risk for humans and animals may be considered. Fortunately, it has been shown that, the use of biolarvicide formulations for example *Bacillus thrungiensis* var. *israeliensis (Bti), Bacillus sphaericus (Bs)* and some insect growth regulators (IRGs) such as pyriproxyfen are effective in controlling malaria vectors. This strategy is cost-effective, feasible, widely accepted by communities, and are safe for use even in domestic water sources and non-target organisms [78]. However, its applications for large habitats such as river streams may need additional investigations.

Current WHO guidelines indicate that larviciding is most appropriate where larval habitats are fixed, few, and findable; and less feasible where habitats are abundant and scattered [79]. While the terms, fixed, few, and findable are often considered finite, it may be better to define them on gradients. This would allow for the determination of the degree to which larval source management may be applicable in different settings. For instance, the findability of habitats, including small or more temporary types could be significantly enhanced by using satellite imagery or unmanned aerial vehicles (UAVs), which enable greater visibility and operational efficiencies [80]. A significant advantage for LSM for *An. funestus* is its reliance on permanent and large aquatic habitats, which are often less numerous than those of other vector species and can persist even in dry seasons [79]. Once identified and characterized, the unique characteristics of these habitats make them potentially easier to target by LSM even in rural areas than the more numerous or expansive habitats of other vector species such as *An. arabiensis*. The relative scarcity and ecological uniqueness of *An. funestus* larval habitats therefore offers excellent opportunities for targeted control. In Tanzania, Nambunga et al. showed that after initial surveys to characterize aquatic water bodies, *An. funestus* habitats in rural settings can fit the description of fixed, few, and findable [46]. In Mexico, where the malaria vector, *Anopheles pseudopunctipennis* also breeds along the river streams like *An. funestus*, the mosquito densities were significantly reduced after implementing an LSM programme involving clearing the vegetation in the sides of the river to expose mosquitoes to sunlight [81]. Controlling *An. funestus* using such an approach, will require defining a comprehensive implementation strategy that integrates community participation to provide the effective workforce needed to operationalize the initiative with maximum impact.

Larval source management was historically one of the most effective malaria control methods but has since been deprioritized in Africa, where methods that target adults, namely ITNs and IRS are now preferred. This was because LSM was considered impractical in African settings due to the abundance of small and temporary larval habitats typically occupied by *An. gambiae s.l.* Such habitats can be difficult to comprehensively locate, characterize and treat promptly. Moreover, the Ross-Macdonald model had further emphasized the significance of reducing adult survival as a more effective approach than reducing vector population size [82]. However, Fillinger & Lindsay have argued against this concept by showing the significance and success of LSM [83]. Some of the best-known examples of historic successes with LSM include the elimination of *An. gambiae* from Brazil and Wadi Haifa, Egypt in the mid-20th century, both of which depended primarily on comprehensive LSM programmes [84]. In recent years, there have been renewed interests in LSM as a supplementary control tool, and many African countries are now including it in their malaria elimination agendas [83]. For example, In Tanzania, following the successful demonstration of LSM impact in urban areas in the mid-2000s [85]), this approach is being promoted in both rural and urban councils to enhance other vector control efforts [85, 86].

The strategic advantage of LSM over IRS and ITNs is that it controls mosquitoes at source [87], and can effectively reduce the population densities of malaria vectors in several settings [83]. LSM could therefore be effective even in areas where mosquitoes are resistant to insecticides used to control adults, or where the adult vector populations are adapted to bite outdoors and/ or on non-human hosts. Effective targeting of habitats used by *An. funestus* is likely to provide a long-term and cost-effective solution, especially if done alongside an adulticiding campaign.

Despite the high potential of LSM in malaria elimination, this approach has some limitations. Larviciding, for example, is currently only recommended in areas where larval habitats are ‘few,‘ ‘fixed,‘ and ‘findable’; often limiting its practical applicability to just the dry seasons since rainfall creates abundant cryptic habitats that may be difficult to treat [79]. On the other hand, habitat modification and manipulation may be unacceptable in certain areas since communities rely on the same habitats for domestic needs (Kahamba et al., unpublished).

### **Targeting adult*Anopheles funestus*using insecticide-treated nets and indoor residual spraying**

Insecticide-treated nets (ITNs) and indoor residual spraying (IRS) have been a major contributor to malaria control since 2000 [2]. Both strategies are increasingly threatened by factors such as insecticide resistance, which affect *An. funestus* as well as other malaria vectors. Studies in Zambia and Tanzania have shown that *An. funestus* populations can survive exposure to pyrethroids at doses up to ten-fold higher than the standard WHO resistance insecticides [88]. Both studies also indicated that the resistance levels in *An. funestus* may be stronger than in the other major vector, such as *An. arabiensis*, in the same locations [88]. Another study in Uganda also showed that *An. funestus* populations were fully resistant to pyrethroids but susceptible to carbamates [89]. It has also been reported in Cameroon that the species is resistant to a range of insecticide classes, including pyrethroids [90]. Resistance in *An. funestus* populations has also been described in west African countries such as Burkina Faso against dieldrin and Benin against DDT [91–93].

Despite having fewer studies on insecticide resistance in *An. funestus* than in *An. gambiae s.l.* [89, 94], a majority of the pyrethroid resistance appears to be of metabolic origin, where the expression of key enzymes such as cytochrome P450 mixed-function oxygenases or glutathione transfereses (GSTs) increase to detoxify pyrethroids and organochlorides such as DDT [95, 96]. So far, no *kdr* mutations have been detected in *An. funestus*. Despite there being significant geographic gaps and relatively limited data on resistance in *An. funestus*, but available information indicates that this vector is extremely resistant to pyrethroids except when co-formulated with PBO synergist; though it is less resistant to non-pyrethroids such as carbamates and organophosphate [34]. The species can also develop multiple resistance mechanisms, and may be more resistant than other malaria vectors [34].

Sustaining the public health value of ITNs and IRS in areas where *An. funestus* dominates, therefore requires improved formulations of existing insecticides or the use of new insecticide classes against which vectors are still susceptible. While these requirements for better insecticide strategies are also needed for other vector species [97], the higher resistance levels in *An. funestus* suggests greater urgency. A range of new vector control have recently become available or are under development with the aim of overcoming resistance in malaria vectors. This includes nets incorporating the synergist, piperonyl butoxide (PBO), and nets with multiple actives including non-pyrethroids which may yield greater benefits if deployed at scale in areas of pyrethroid resistance [98, 99]. In line with current WHO guidelines on PBO nets, most of the east and southern Africa region already have moderate to strong resistance and would qualify for PBO net distribution [100]. Unfortunately, the majority of these new products have so far been evaluated against only *An. gambiae s.l*, thus there is need to understand how they might affect *An. funestus* populations. However, in northern Tanzania districts where *An. funestus* was the dominant malaria vector, ITNs with multiple actives have recently demonstrated superior performance over pyrethroid-only ITNs, clearly illustrating the potential of such innovations [101].

Similarly, the efficacy of IRS for *An. funestus* control could be improved through the use of longer lasting formulations based on non-pyrethroid insecticides. Unlike ITNs, which are primarily dependent on pyrethroids, IRS campaigns have largely phased out pyrethroids and are now done using either carbamates, organophosphates, or neonicotinoids [102]. IRS impact depends on consistent application of high-quality insecticides, with spraying done at preferably twice yearly, and repeated for several years until malaria transmission intensities drop below locally acceptable thresholds [100]. IRS has been particularly effective against indoor resting malaria vectors including *An. funestus* [103], with the highest impact for malaria control occurring in rural Africa. For instance, early evidence from Tanzania indicated that after a period of spraying in Pare and Taveta region, IRS effectively eliminated local populations of *An. funestus* with no re-colonization for at least eight years [104].

This sustained impact was achieved because of the highly endophilic behaviour of *An. funestus*, coupled with the scarcity and dispersed nature of suitable larval habitats which slowed local re-colonization once the vector populations started dwindling. Similarly, evidence from southern Africa where IRS with DDT was widely implemented indicates this approach successfully contained transmission by *An. funestus* over five decades [3, 96]. When the programme transitioned to pyrethroids instead of DDT between 1997 and 1999, populations of *An. funestus* carrying pyrethroid-resistance reinvaded the areas causing new malaria epidemics in 2000 and prompting the reinstatement of DDT [105, 106].

Taken together, this evidence suggests that a consistent programme of adulticiding with carefully selected insecticides against which the vector is susceptible could dramatically crash malaria transmission in areas where *An. funestus* is dominant. Based on this hypothesis, a simplified approach for high-quality and high-coverage IRS or other forms of adulticiding would have a disproportionately impact and perhaps result in reducing *An. funestus* populations in a given area. The impacts would be amplified if the intervention targeting adults is accompanied by an effective LSM programme that targets the right kind of aquatic habitats, hence reducing the likelihood of re-colonization of the areas and sustaining the gains.

Other than insecticide resistance, another important concern regarding IRS is that it can be logistically difficult and expensive to implement in large scale. In fact, while the number of countries adopting IRS has increased since 2000, the number of people protected appears to stagnate, as the countries adopt more targeted and small-scale operations. Other challenges include the high quantities of insecticides necessary, the need for large spray teams that are well-trained, challenges with disposal of unused pesticides and pesticide wastes and the need to remove household belongings during spraying. It is important, therefore, that future efforts should target improved formats for delivering IRS or its equivalents in ways that do not compromise the public health value.

### **Other interventions with potential against*Anopheles funestus*adults**

In addition to the proposed strategic use of IRS, ITNs, and LSM, vector control against *An. funestus* could benefit from additional interventions targeting adults during different life-history stages or behaviours. To be most efficacious, selection of the complementary interventions must be informed by basic understanding of the natural attributes of the vector species. One example could be the use of attractive targeted sugar baits (ATSBs), which kill mosquitoes during sugar feeding. This intervention has the benefit of being usable both indoors and outdoors, and being able to target both male and female mosquitoes [70]. Recent field observations of *An. funestus* males occurring at high frequencies indoors suggest that males could be readily targetable by ATSBs or other indoor approaches [107].

Other options that could effectively reduce exposure to *An. funestus* are house improvements such as house screening [108] and eave-based interventions, which target mosquitoes when entering houses through the eave spaces. In particular, the eave-based interventions may include insecticide-treated eave ribbons [109], eave baffles [110] and eave tubes [111]. These interventions have the additional advantage of being less cumbersome than IRS and requiring far lower quantities of insecticides. Importantly, because the eave spaces are distally removed from human contact, a much wider range of insecticide classes could be used on these interventions, preferably those which have no cross-resistance with pyrethroids. Such house-based approaches are anticipated to be particularly effective against *An. funestus* given its highly endophilic and endophagic nature.

There are also non-insecticidal interventions that may be effective for *An. funestus* control. For example, mass deployment of odor-baited traps on Rusinga Island in western Kenya resulted in more than 40% reduction in malaria incidence, primarily by targeting *An. funestus* [107]. Mathematical simulations suggest that odor baited traps used alongside ITNs could significantly improve control and potentially lead to local elimination in multiple settings across Africa [112, 113].

It has been proposed that genetically modified mosquitoes carrying the gene drive technology could also eventually be an alternative to broadly address current challenges with vector control. However, current gene drive developments for malaria control are primarily focused on *An. gambiae s.s.* [114, 115] and have no immediate applications in areas dominated by *An. funestus*. However, recent work has suggested that certain types of gene drives, which employ homology-directed repairs to ensure their proliferation in the genomes may be suitable for use in *An. funestus* [116]. Along with further advancements in genetic technology, a deeper knowledge of the mating behaviour and gene flow trajectories in this species will be critical for evaluating the potential for such genetic approaches in controlling *An. funestus*. Since the public health value of the above alternative tools has not yet been confirmed, additional research is necessary to determine their true potential and cost-effectiveness.

### **Community engagement to enhance the control of malaria in areas dominated by*Anopheles funestus***

To ensure the success of existing or novel interventions for *An. funestus* control, it is crucial to engage community members and other key stakeholders when planning the implementation of these interventions [117]. Early and continuous community engagement is vital in guaranteeing usability, acceptability, sustainability, and overall effectiveness of the interventions [117]. Community members generally have significant levels of knowledge and experiences, which can be valuable in ensuring success of malaria control interventions. Detailed qualitative surveys may be necessary to understand these community views and the potential acceptability of any treatment or manipulation of the aquatic habitats. For best results, the community engagement initiatives should go beyond simply raising awareness about a particular intervention. Instead, the initiatives should also build partnerships with the communities to create and/or improve their sense of ownership of the interventions; and to encourage their participation in the success of the interventions [118].

There are numerous documented ways to engage the communities in malaria control efforts in Africa. In southern Tanzania, Mwangungulu et al. demonstrated that community members could be relied upon to identify areas with the highest densities of malaria vectors, a useful means for low-cost community-based planning of malaria control [119]. Other studies in Tanzania and Burkina Faso have also demonstrated that community members can be relied upon to identify and spray *Anopheles* mosquito swarms with insecticides [66, 120]. Additionally, household members were recruited to monitor human activities and behaviours that increase the risk of contact with malaria vectors [121].

It has been observed that important *An. funestus* habitats, such as spring-fed pools, ponds, and streams, often also serve as water sources for domestic uses, irrigation, or livestock use (Kahamba et al., pers. commun.). In this regard, local communities can be involved to integrate LSM into their daily practices. Such strategies have already been demonstrated on a small scale in rural Tanzania, where pastoralists were recruited to identify and treat aquatic mosquito habitats during the dry season [122]. A related example is where larvicides have been mixed with fertilizers so that farmers could apply these to their farms to provide the added advantage of mosquito control. Such programmes could be expanded and improved by training selected members of local communities to identify and treat potential habitats for *An. funestus*.

Lastly, for community members to have meaningful involvement in malaria control efforts, they must have good awareness and understanding of the risk, burden, and severity of malaria. Improving a community’s knowledge and awareness needs to go beyond merely explaining scientific knowledge to the community members. It must also consider important cultural values, experiences, practices and interests in the respective communities [117].

## Conclusions

*Anopheles funestus* is widely distributed and accounts for a higher proportion of malaria transmission in East and South African countries. While research on this species has been limited partly due to difficulties in creating laboratory colonies, available evidence suggests it possesses several distinct ecological characteristics which may render it amenable to certain high-impact interventions approaches targeting both its immature and adult stages. Its preferred aquatic habitats tend to be few and non-temporary and may include rivers, streams, large ponds, and spring-fed pools. This species is mostly endophilic and anthropophagic though both outdoor-feeding and animal-biting populations have also been reported, especially where residents keep a lot of livestock. The existence and magnitude of these “atypical” behaviours need to be considered when designing complementary interventions. Considering the dominance and ecological distinctiveness of *An. funestus*, it is hypothesized that combining targeted larval source management and at least one method that effectively target adults (including insecticide-resistant populations) could be both operationally feasible and highly impactful. In areas where *An. funestus* is the dominant vector, the approach could cause major reductions in malaria transmission by drastically reducing the local populations of the species and limiting the likelihood of its re-colonization. For best results, the programme may be followed by gradual house screening to maintain a low-level transmission and cultivating strong community engagement to guarantee sustainability. It should also be recognized that the broader goal of malaria elimination would require a much more expansive operation targeting all important vectors beyond *An. funestus.*

## Data Availability

Not applicable.
